# FKBP10 promotes proliferation of glioma cells via activating AKT-CREB-PCNA axis

**DOI:** 10.1186/s12929-020-00705-3

**Published:** 2021-02-09

**Authors:** Hong-Qing Cai, Min-Jie Zhang, Zhi-Jian Cheng, Jing Yu, Qing Yuan, Jin Zhang, Yan Cai, Li-Yan Yang, Yu Zhang, Jia-Jie Hao, Ming-Rong Wang, Jing-Hai Wan

**Affiliations:** 1 Department of Neurosurgery, National Cancer Center/National Clinical Research Center for Cancer/Cancer Hospital, Chinese Academy of Medical Sciences and Peking Union Medical College, Beijing, China; 2 State Key Laboratory of Molecular Oncology, Center for Cancer Precision Medicine, National Cancer Center/National Clinical Research Center for Cancer/Cancer Hospital, Chinese Academy of Medical Sciences and Peking Union Medical College, Beijing, China; 3 Department of Neurosurgery, The Second Affiliated Hospital, Anhui Medical University, Hefei, China; 4 Department of Neurosurgery, Beijing Tiantan Hospital, Capital Medical University, Beijing, 100070 China

**Keywords:** Glioma, Proliferation, FKBP10, AKT, CREB, PCNA

## Abstract

**Background:**

Although the availability of therapeutic options including temozolomide, radiotherapy and some target agents following neurosurgery, the prognosis of glioma patients remains poor. Thus, there is an urgent need to explore possible targets for clinical treatment of this disease.

**Methods:**

Tissue microarrays and immunohistochemistry were used to detect FKBP10, Hsp47, p-AKT (Ser473), p-CREB (Ser133) and PCNA expression in glioma tissues and xenografts. CCK-8 tests, colony formation assays and xenograft model were performed to test proliferation ability of FKBP10 in glioma cells in vitro and in vivo. Quantitative reverse transcriptase-PCR, western-blotting, GST-pull down, co-immunoprecipitation and confocal-immunofluorescence staining assay were used to explore the molecular mechanism underlying the functions of overexpressed FKBP10 in glioma cells.

**Results:**

FKBP10 was highly expressed in glioma tissues and its expression was positively correlates with grade, poor prognosis. FKBP10-knockdown suppressed glioma cell proliferation in vitro and subcutaneous/orthotopic xenograft tumor growth in *vivo*. Silencing of FKBP10 reduced p-AKT (Ser473), p-CREB (Ser133), PCNA mRNA and PCNA protein expression in glioma cells. FKBP10 interacting with Hsp47 enhanced the proliferation ability of glioma cells via AKT-CREB-PCNA cascade. In addition, correlation between these molecules were also found in xenograft tumor and glioma tissues.

**Conclusions:**

We showed for the first time that FKBP10 is overexpressed in glioma and involved in proliferation of glioma cells by interacting with Hsp47 and activating AKT-CREB-PCNA signaling pathways. Our findings suggest that inhibition of FKBP10 related signaling might offer a potential therapeutic option for glioma patients.

## Background

Glioma accounts for about 70% of all primary malignant brain tumors. According to the classification criteria of World Health Organization, glioma is subtyped into low grade (LGG, grade 1–3) and high grade (HGG, grade 4) [1]. Although Stupp protocol (radiotherapy plus concomitant and adjuvant temozolomide) following neurosurgery have shown high tumor control rate [2, 3], the prognosis of glioma patients remains poor, the 5-year overall survival rates for LGG and HGG are about 25% and 5%, respectively [4]. Thus, there is an urgent need to identify molecule alterations involved in malignant phenotypes of glioma cells and to explore possible targets for clinical treatment of this disease.

FKBP is a protein family with affinity for FK506, a compound with immunosuppressant. FKBP proteins are involved in multiple cellular processes, including receptor signaling, protein folding, chaperone activity, transcription and immunosuppression [5]. Several FKBPs have been implicated in biological behavior of cancer cells. For example, FKBP52 binding to heat shock protein 90 enhanced androgen receptor function and promoted proliferation of prostate cells [6]. FKBP3, FKBP4 and FKBP8 maintained the ability of proliferation and migration/invasion of lung and breast cancer cells [7–9]. FKBP51 increased PHLPP-AKT interaction and facilitated PHLPP-mediated dephosphorylation of AKT at Ser473, thus downregulating AKT activation in pancreatic cancer cells [10].

FKBP10 is a member of FKBP family and encodes a 65-KD protein (also termed FKBP65). FKBP10 protein is located in rough endoplasmic reticulum and involved in collagen biosynthesis through collagen pyridinoline cross-linking and construction of bone, tendons [11, 12]. Hypofunction of FKBP10 protein induced by FKBP10 mutation reduced collagen formation, promoted bone fragility or joint contracture, and thus caused several severe diseases such as osteogenesis imperfect, Bruck syndrome [13, 14]. FKBP10 overexpression was found in lung fibroblast of idiopathic pulmonary fibrosis (IPF), leading to an impact on extracellular matrix protein synthesis and secretion [15, 16]. Nintedanib approved for idiopathic pulmonary fibrosis therapy significantly could down-regulate FKBP10 expression in IPF fibroblasts [17]. Besides, FKBP10 upregulation has been observed in KRAS mutation-induced lung adenocarcinoma, colorectal cancer renal cell carcinoma, gastric cancer and knockdown of FKBP10 expression could reverse the malignant phenotype, especially proliferation ability [18–21]. Thus, FKBP10 is an important molecule in cell biology activities and a potential therapeutic target with therapeutic drug. However, biological role of FKBP10 in glioma remains unclear.

In the present study, we found that FKBP10 was upregulated in glioma. High FKBP10 expression was an independent indicator for poor prognosis of grade 4 glioma patients. Moreover, FKBP10 promoted proliferation of glioma cells through AKT-CREB-PCNA signaling and might be as a possible therapeutic target for glioma.

## Materials and methods

### Glioma tissues

A total of 430 glioma tissues were obtained from the Department of Neurosurgery at the Cancer Hospital, Chinese Academy of Medical Sciences. Thirty specimens had matched adjacent non-neoplastic tissues collected from the edema-affected tissues surrounding the high-grade gliomas or resected in the process of obtaining deep-seated gliomas. Two independent neuropathologists diagnosed all the specimens according to the WHO classification. Grade 2, 3 and 4 gliomas were of 17.2%, 16.3% and 66.5%, respectively. All the enrolled patients were without history of other malignant tumors and received neither neoadjuvant chemotherapy nor radiotherapy before surgery, and signed separate informed consent forms for the sampling and molecular analyses. The clinical/pathological information of the patients are shown in Additional file 1: Table S1, in which the follow-up information was available for 394 patients. All the operative specimens were residual tissues after diagnostic sampling. This study was approved by the Ethics Committee of Cancer Hospital, Chinese Academy of Medical Sciences (No. NCC2014G-12).

### Immunohistochemistry

The cores of operative tissues and glioma cell lines were extracted from the primary blocks to construct tissue microarrays (TMAs). TMAs and paraffin-embedded subcutaneous xenograft were cut into 5-μm sections. Immunohistochemistry and evaluation of immunostaining were performed as described previously [22]. The following primary antibodies were used: anti-FKBP10 antibody (1:2000, 50353, Sigma), anti-Hsp47 antibody (1:1200, sc5293, Santa Cruz), anti- p-CREB (1:1000, 9198S, CST) and PCNA (1:5000, 13110S, CST). PV-9000 Polymer Detection System (PV9000, ZSGB-BIO) following the manufacturer’s instructions was used to visualize the immune-staining.

### Cell culture and inhibitors treatment

The human glioma cell lines GOS-3, T98G, U118MG, LN229, U251MG, SF268, U343MG, U373MG, HS683 and TJ905 were purchased from the Cobioer Biotechnology Company (Nanjing, China) and National Infrastructure of Cell Line Resource (Beijing, China). All cells were cultured in high-glucose DMEM except for SF268 in 1640 and T98G in MEM with containing 10% fetal bovine serum, 100 mg/mL streptomycin, and 100 U/mL penicillin and 73 maintained in a humidified atmosphere containing 5% CO2 at 37 °C. The cell lines were incubated with the p-Akt activator SC79, p-CREB inhibitor KG-501 and MG132 (Selleck) for different lengths of time.

### siRNA and shRNA construction

The used siRNAs were as follows: 5′-CTACCACTACAACGGCACTTT-3′ (FKBP10-siRNA1), 5′-GAAGATTATCATCCCTCCATT-3′ (FKBP10-siRNA2), 5′-CAGCAGC AAGCAGCACTACAA-3′ (Hsp47-siRNA1), 5′-CAACTACTACGACGACGAGAA- 3′ (Hsp47-siRNA2). Non-silencing siRNA as control was 5′-TTCTCCGAACGTGTCA CGT-3′. The target sequence of the FKBP10 shRNA was 5′-GCTCTCATCTTGCTC A ATTCC-3′, and the scramble was 5′- GGATCATCATGCTATGCAGTT-3′. All targeted sequences were synthesized by GenePharma (Suzhou, China).

### Transfection and lentiviral transduction

Cells were transfected with siRNA using Lipofectamine 2000 (Life Technologies, Carlsbad, CA, USA) based on the manufacturer’s instructions. The final concentration of siRNA used for gene silencing is 100 nM. Cells were collected for subsequent analyses after 48 h post-transfection. The lentiviruses were used to transduce glioma cells, and stable cell strains expressing FKBP10-shRNA (shFKBP10) or control scramble-shRNA (sh-scramble) were selected by using puromycin (2 μg/mL, Gibco) for at least 1 week.

### Western blot analysis

Total protein was isolated using RIPA buffer (Applygen, Beijing, China) with protease inhibitors and phosphatase inhibitors (Roche, Basel, Switzerland) according to the manufacturers’ instructions. Western blot was performed by routine operation. Immunoblotting was carried out with primary antibodies against FKBP10 (1:500, 50353, Sigma), Hsp47 (1:500, sc-5293, Santa Cruz), p-AKT (Ser473) (1:1000, 4060S, CST), Akt (1:1000, 2920S, CST), p-CREB (1:1000, 9198S, CST), CREB (1:1000, 9197S, CST), PCNA (1:1000, 13110S, CST). GAPDH (1:5000, 60004-1-Ig, Proteintech) was used as a loading control. Secondary antibodies (Goat anti-Mouse IgG and Goat anti-Rabbit IgG, 1:5000) were purchased from Applygen. The signals were visualized with a super enhanced chemiluminescence (ECL) detection reagent (Applygen).

### RNA extract and real-time PCR

Total RNA was isolated using an RNApure Tissue & Cell Kit (Cwbiotech, Beijing, China). The isolated RNA was used as a template for reverse transcription reactions using a HiFiScript cDNA Synthesis Kit (Cwbiotech). Quantitative real-time RT-PCR analysis was performed using SYBR® Fast qPCR Mix (TaKaRa, Shiga, Japan) and a CFX96 Real-Time System (Bio-Rad). The relative mRNA expression of the target genes was normalized to an endogenous reference (GAPDH). The primer sequences used in this study were listed as follows: PCNA F 5ʹ-GCCCTGGTTCTGGAGGTAA C-3ʹ, R, 5ʹ-GTCCTTGAGTGCCTCCAACA-3ʹ; GAPDH F, 5ʹ-ATTCCATGGCACCG TCAAGGCTGA-3ʹ, R, 5ʹ-TTCTCCATGGTGGTGAAGACGCCA-3ʹ.

### Co-immunoprecipitation assay

Total protein was isolated from cells using a non-denaturing lysis buffer (Applygene) with protease inhibitors. Protein G agarose beads were incubated with anti-FKBP10 antibody, anti-Hsp47 antibody, rabbit or mouse IgG at room temperature for 1 h. Then, the protein lysate were added and incubated at 4 °C overnight. The immunoprecipitates were collected by centrifugation and washed with PBST. The mixture was subjected to western blot analysis.

### Immunofluorescence microscopy

The cells that grew on the slides were fixed, permeabilized, blocked and incubated with FKBP10 (1:100), Hsp47 (1:100) at 4 °C overnight. The bounding primary antibodies were detected using goat anti-mouse IgG-FITC or goat anti-rabbit IgG H&L (1:100) (Abcam) at 37 °C for 1 h. The fluorescence was detected via confocal microscopy (General Electric Company, Fairfield, CT, USA).

### Construction of FKBP10 mutant glioma cells

pEGFP-C1 plasmid with full length cDNA of FKBP10 were used to construct FKBP10 domain deletion mutants according to KOD-Plus-Mutagenesis Kit’s instructions (SMK-101, TOYOBO). The primer sequences used were listed in Table 1. The five deletion mutants were transfected separately into shFKBP10 LN229 cells.

**Table 1:** The primer sequences for constructing FKBP10 domain deletion mutants

FKBP10 mutants	Forward	Reverse
∆1 (aa1–150)	AACAAGGAAGACACCGTGCAGGTGAGCACAT	CATAGAATTCGAAGCTTGAGCTCGAGATC
∆2 (aa174–262)	AACCCGAAGGACGCTGTCCAGCTAGAGAC	GTCCTGGACCATGCGGGGGCAGTG
∆3 (aa286–374)	AACCCTGCGGATGTGGTGGAAATCAGGACAC	CCCGGCCCCGGCTCTGCGGACA
∆4 (aa399–486)	GATGGGCTGCCCACAGGCTACCTGTTTG	AAGCTTGGTGGTCTCATTGCAGGTCTCAGATGG
∆5 (497–582)	GGATCCACCGGATCTAGATAACTGATCATAAT	CACAAACAGGTAGCCTGTGGGCAGCCC

### Cell viability and colony formation assay

Cells were seeded in triplicate in 96-well plates at a density of 2000 cells per well. Cell viability was assessed with a Cell Counting kit-8 (CCK-8, Dojindo, Japan) according to the manufacturer’s protocol. The absorbance at 450 nm was measured using an ELX808 microplate spectrophotometer (BioTek Instruments, Winooski, VT, USA). To assess the clonogenic capacity of cells in vitro, 500 cells were plated in six-well culture plates in triplicated. The cells were allowed to grow for 10–14 days to form colonies, and then fixed with methanol and stained with crystal violet. The number of colonies was counted.

### In vivo* study*

All animal experiment procedures were in accordance with acquirements of Experimental Animal Ethics Committee and approved by the Animal Center of the Institute of National Cancer Center/Cancer Hospital, CAMS & PUMC (NCC2019A014). For subcutaneous xenograft model, 10^6^ cells of FKBP10-shCtrl and FKBP10-shRNA LN229 cells were subcutaneously injected in 4–5 week-old female BALB/c-nude mice (n ≥ 5 mice each group) separately. The tumor size was weekly measured, and the mice were sacrificed after 6 weeks. The tumor specimens were embedded in paraffin, cut into 5-μm sections, and stained with hematoxylin and eosin (H&E). For orthotopic model, 5 × 10^5^ cells in 5 μL were subcutaneously injected into the caudate nucleus of brain of 4–5 week-old female BALB/c-nude mice (n ≥ 3 mice each group) separately with continuous anesthetization with isoflurane (RWD Life Science). Then orthotopic xenograft were tested with T2-weight (T2W) by a 7.0 T MRI scanner (Bruker BioSpin, Billerica, MA, USA) after 4 weeks.

### Statistical analysis

All statistical analyses and drawing figures were performed by using R software and GraphPad Prism software (GraphPad Inc., San Diego, USA). The survival analysis was done with “survival” and “survminer” package. The correlation analysis was done by Spearman correlation test. The experimental results were statistically evaluated using Student’s t-test, one-way ANOVA, or the nonparametric Kruskal–Wallis test for comparisons between different groups. *P* < 0.05 was considered statistically significant.

## Results

### FKBP10 overexpression was positively related to glioma grade and negatively related to survival time

**Fig. 1:**
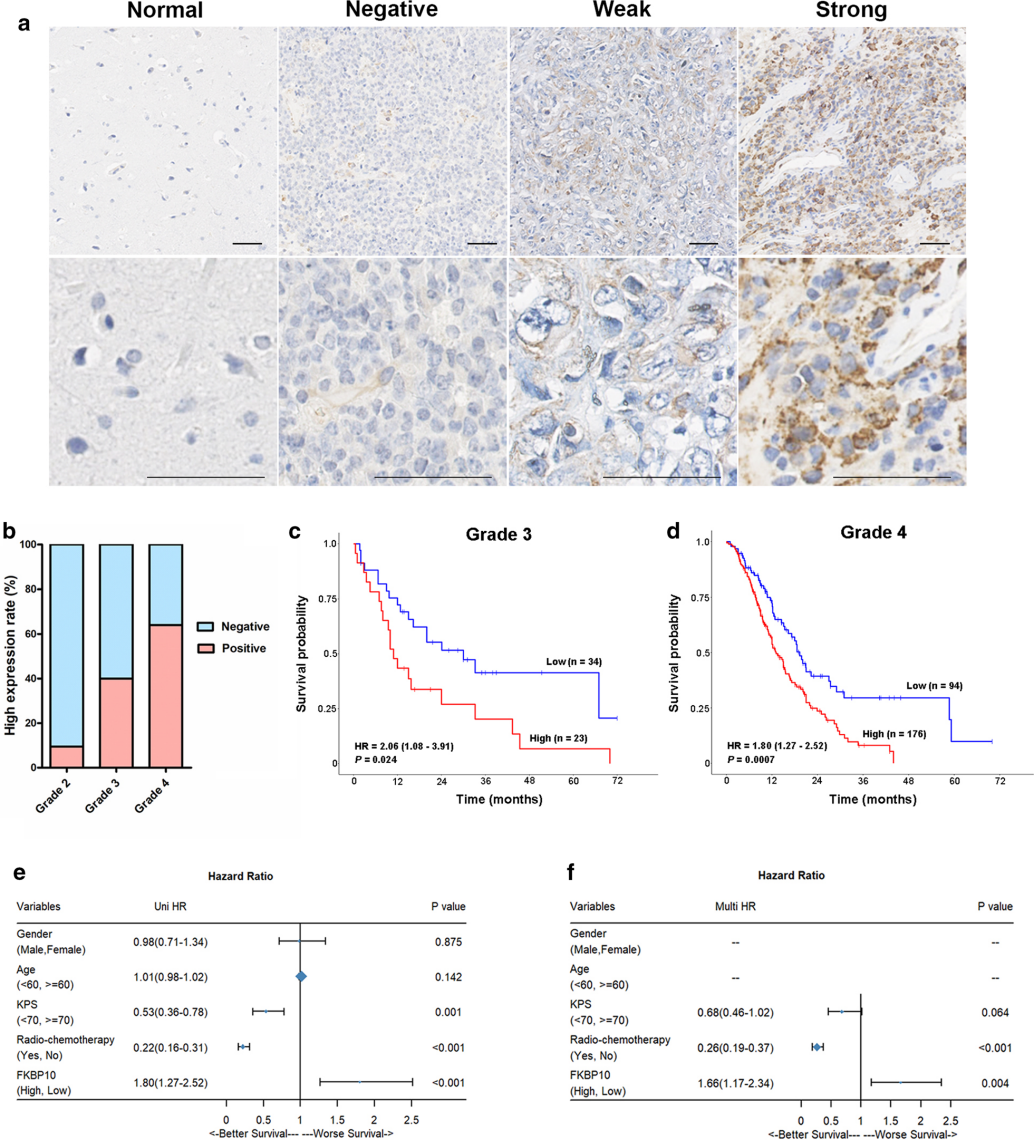
The clinical value of FKBP10 expression in glioma tissues. **a** Representative images of FKBP10 immunostaining in non-neoplastic tissues and glioma tissues. Scale bar = 100 μm. **b** FKBP10 expression in tumors of grades 2, 3 and 4. **c**, **d** Survival analysis of FKBP10 overexpression in tumors of grades 3 and 4. **e**, **f** Forest plot depicting the results of univariate and multivariate analysis of gender, age, KPS, Radio-chemotherapy and FKBP10 expression in 280 gliomas of grade 4

Immunochemistry analysis showed that FKBP10 was undetectable in all the tested non-neoplastic tissues, but strongly expressed in 5.4%, 40.0% and 64.7% of grades 2, 3, 4 gliomas, with an increasing frequency in higher grade tumors (Fig. 1a, b). In tumor tissues, FKBP10 protein was located in the cytoplasm of cancerous cells. Strong FKBP10 immunostaining was positively related to poor prognosis in both grades 3 (HR = 2.06, 95% CI 1.08–3.91, *P* = 0.024) and 4 gliomas (HR = 1.80, 95% CI 1.27–2.52, *P* = 0.0007, Fig. 1c, d). Multivariate Cox regression analysis indicated that FKBP10 overexpression was an independent indicator for poor prognosis of grade 4 glioma patients (Fig. 1e, f).

### Reduced FKBP10 expression inhibited proliferation of glioma cells

**Fig. 2:**
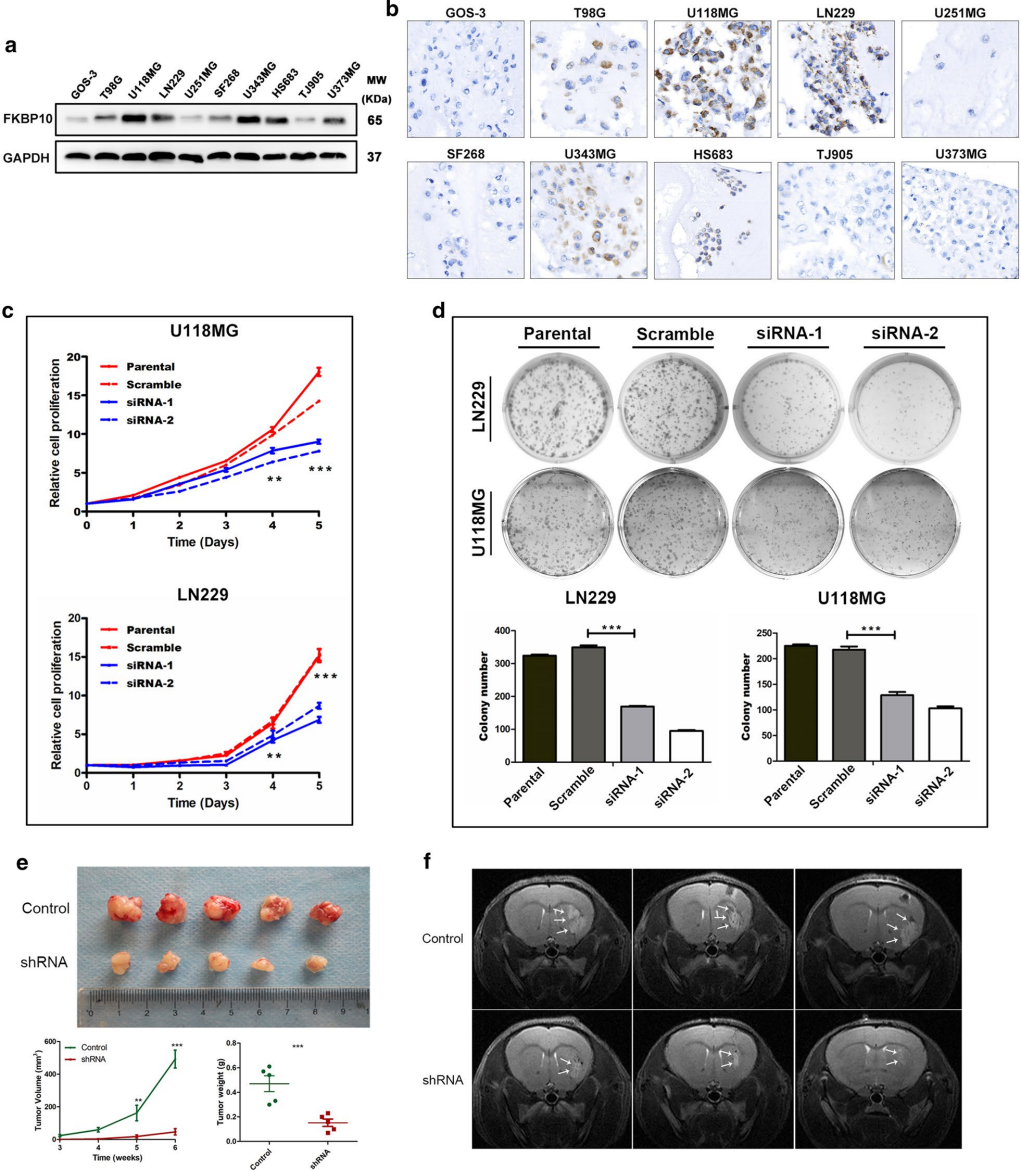
Downregulation of FKBP10 reduced proliferation of glioma cells in vitro and in vivo. **a**, **b** Western blot analysis and immunocytochemistry assays of FKBP10 expression in 10 glioma cell lines. **c** The results of CCK-8 cell proliferation tests in FKBP10 parental, scramble, siRNAs groups of U118MG and LN229 cells. **d** Representative images of colony formation assays in FKBP10 parental, scramble, siRNAs groups of U118MG and LN229 cells. Statistical results of colony number. **e** Image of subcutaneous tumors in nude mice. Statistical results showed that tumor volume and weight were significantly decreased in FKBP10 shRNA group. **f** Images of T2-weighted MRI showed intracranial xenografts of FKBP10-shRNA group grew slower than that of control group. The data are represented as means ± SD. **P < 0.01, ***P < 0.001

Western-blot and immunocytochemistry analyses of 10 glioma cell lines showed that high FKBP10 expression was in T98G, U118MG, LN229, U343MG and HS683, and located in cellular cytoplasm (Fig. 2a, b). We therefore chose U118MG and LN229 cell lines for the subsequent exploration. Knockdown of FKBP10 expression in glioma cells led to a significant reduce of cell proliferation rate and colony number compared to the control cells (Fig. 2c, d). In vivo experiments showed that knockdown of FKBP10 in LN229 glioma cells significantly decreased the tumorigenic ability. Subcutaneous tumors with FKBP10-shRNA LN229 cells were of markedly reduced volumes and weights (Fig. 2e). Intracranial xenografts derived from FKBP10-shRNA cells had smaller tumors than those of the control, as revealed by T2-weighted MRI (Fig. 2f).

### FKBP10 affects AKT-CREB signal and PCNA expression in glioma cells

**Fig. 3:**
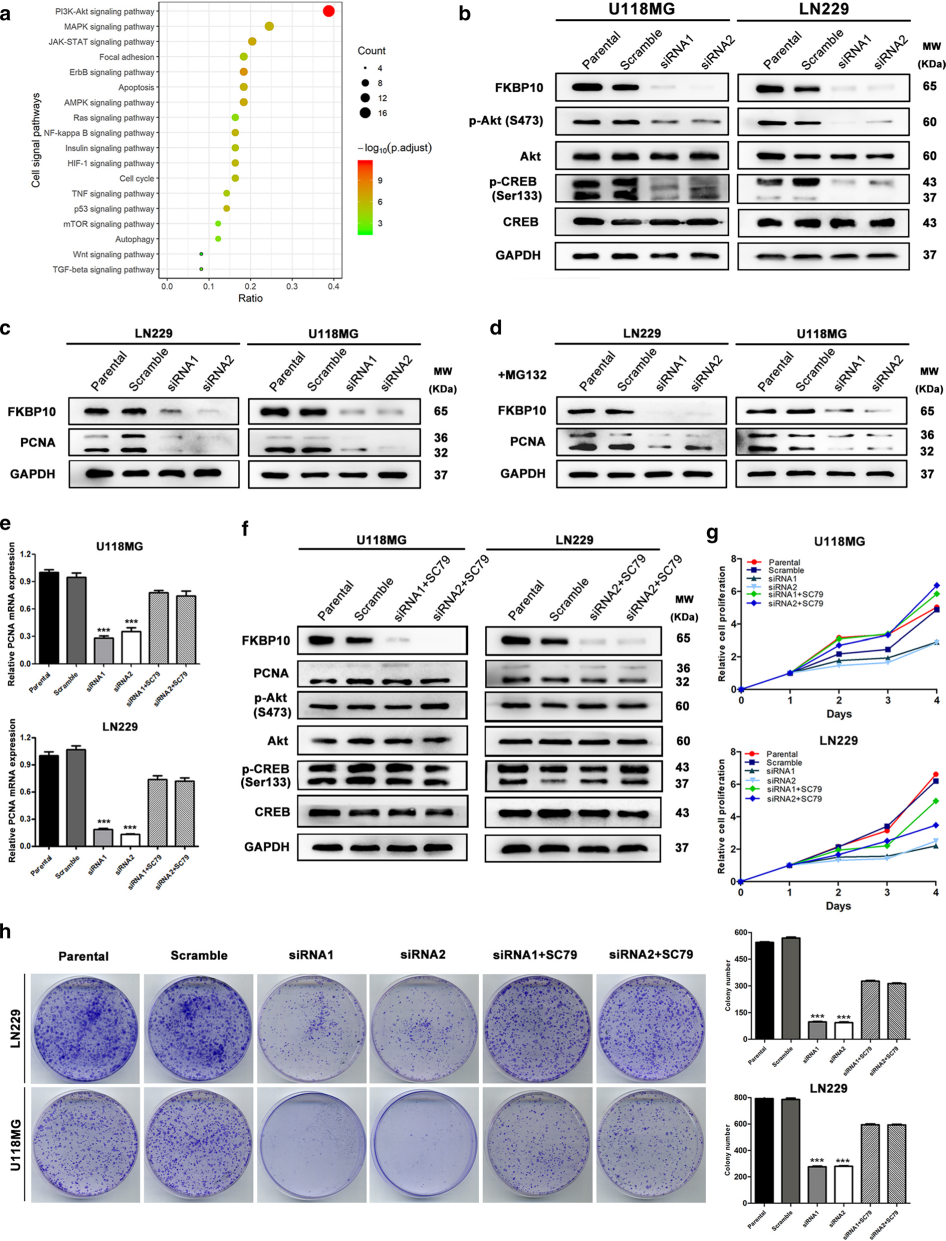
FKBP10 related molecules and signaling pathways. **a** Pathway analysis of the differentially phosphoproteins and unphosphoproteins in FKBP10 siRNA LN229 compared with control cells. **b** The expression level of AKT, CREB and their phosphoproteins in parental, scramble, siRNAs groups of U118MG and LN229 cells. **c**, **d** PCNA protein expression level in FKBP10 knockdown and non-silencing cells with or without incubating with proteasome inhibitor MG132. **e** PCNA mRNA levels were analyzed using real-time RT-PCR. **f**–**h** The reduced p-AKT, p-CREB, PCNA expression and proliferation ability were restored in FKBP10 knocdown glioma cells through incubating with AKT activator SC79. The data are represented as means ± SD. *P < 0.05, **P < 0.01, ***P < 0.001

We checked a total of 157 phosphoproteins and 147 unphosphoproteins playing vital role in 16 cell signal patyways by phospho-specific protein microarray. Significantly upregulated and downregulated proteins were enriched in PI3K-AKT pathway, followed by MAPK and JAK-STAT signal pathways (Fig. 3a). Western blot analysis showed that phospho-AKT (Ser473) and phospho-CREB (Ser133) were significantly downregulated after transfection with FKBP10-siRNA in U118MG and LN229, while there were no change in total protein levels of AKT and CREB (Fig. 3b). Previous study suggested that Proliferating cell nuclear antigen (PCNA) plays an essential role in the proliferation process. We found PCNA expression was also reduced in FKBP10-knockdown cells, which was not reversed after treated with MG132 for 12 h (Fig. 3c, d). Real-time PCR assay detected an obvious decrease of PCNA mRNA exprssion following by FKBP10-knockdown (Fig. 3e), which suggested that FKBP10 motivates PCNA protein expression dependent on upregulation of transcript process. When treated FKBP10-knockdown cells with AKT activator SC79 0.25 mol/L for 24 h, the reduced p-AKT, p-CREB, PCNA expression, and the proliferation ability of FKBP10-knockdown cells were apparently rescued (Fig. 3f–h).

### FKBP10 promotes AKT phosphorylation by interacting with Hsp47

**Fig. 4:**
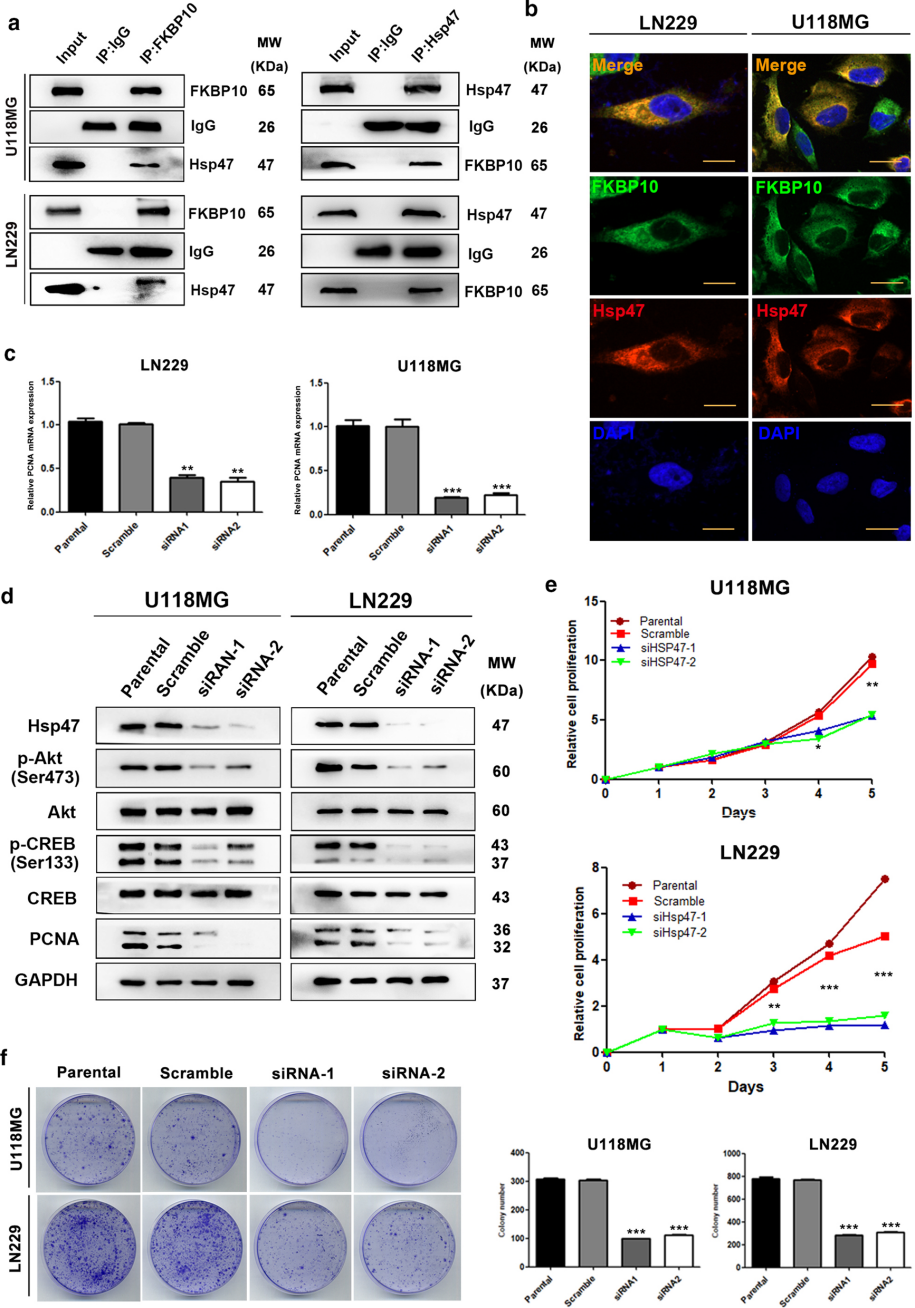
Hsp47 interacting with FKBP10 enhanced the proliferation phenotype. **a** Interaction between FKBP10 and Hsp47 in glioma cells was detected by immunoprecipitation-Western blot assay. **b** Cellular localization of FKBP10 and Hsp47 was detected by immunofluorescence staining. DAPI was used to stain nuclei (blue). Scale bar = 30 μM. **c**, **d** The expression level of PCNA mRNA, PCNA protein, AKT, p-AKT, CREB, p-CREB and cell proliferation in Hsp47 parental, scramble, siRNAs groups of U118MG and LN229 cells. **e** Cell proliferation tests in Hsp47 parental, scramble, siRNAs groups of U118MG and LN229 cells by CCK-8 assays. **f** Representative images of colony formation assays in Hsp47 parental, scramble, siRNAs groups of U118MG and LN229 cells. Statistical results of colony number. The data are represented as means ± SD. *P < 0.05, **P < 0.01, ***P < 0.001

GST-pull down and liquid chromatography–mass spectrometry (LC–MS)/MS analyses showed that heat shock protein 47 (Hsp47) was preferentially bind to FKBP10 in glioma cells. Co-immunoprecipitation assay (Co-IP) assay indicated that FKBP10 probably interacted with Hsp47 in U118MG and LN229 cells (Fig. 4a). Immunofluorescence assay demonstrated that FKBP10 was co-located with Hsp47 (Fig. 4b). We then tested the Hsp47 expression in 10 glioma cell lines, and observed high Hsp47 protein expression in U118MG and LN229 cells (Additional file 2: Fig S1A, B). In consistent with that of FKBP10, knockdown of Hsp47 also led to a significant reduce of PCNA mRNA and protein, p-AKT (Ser473), p-CREB (Ser133) expression (Fig. 4c, d), as well as cell proliferation rate and colony number (Fig. 4e, f). Nevertheless, both FKBP10 and Hsp47 had no regulatory relationship with each other (Additional file 2: Fig S1C, D). All these results suggest that FKBP10 interacted with Hsp47, promoted the phosphorylation of AKT and CREB, upregulated the expression of PCNA mRNA and PCNA protein level, and thus enhanced the proliferation ability of glioma cells.

### The FKBP-type3 PPIase domain is an active domain of FKBP10 interacting with Hsp47

**Fig. 5:**
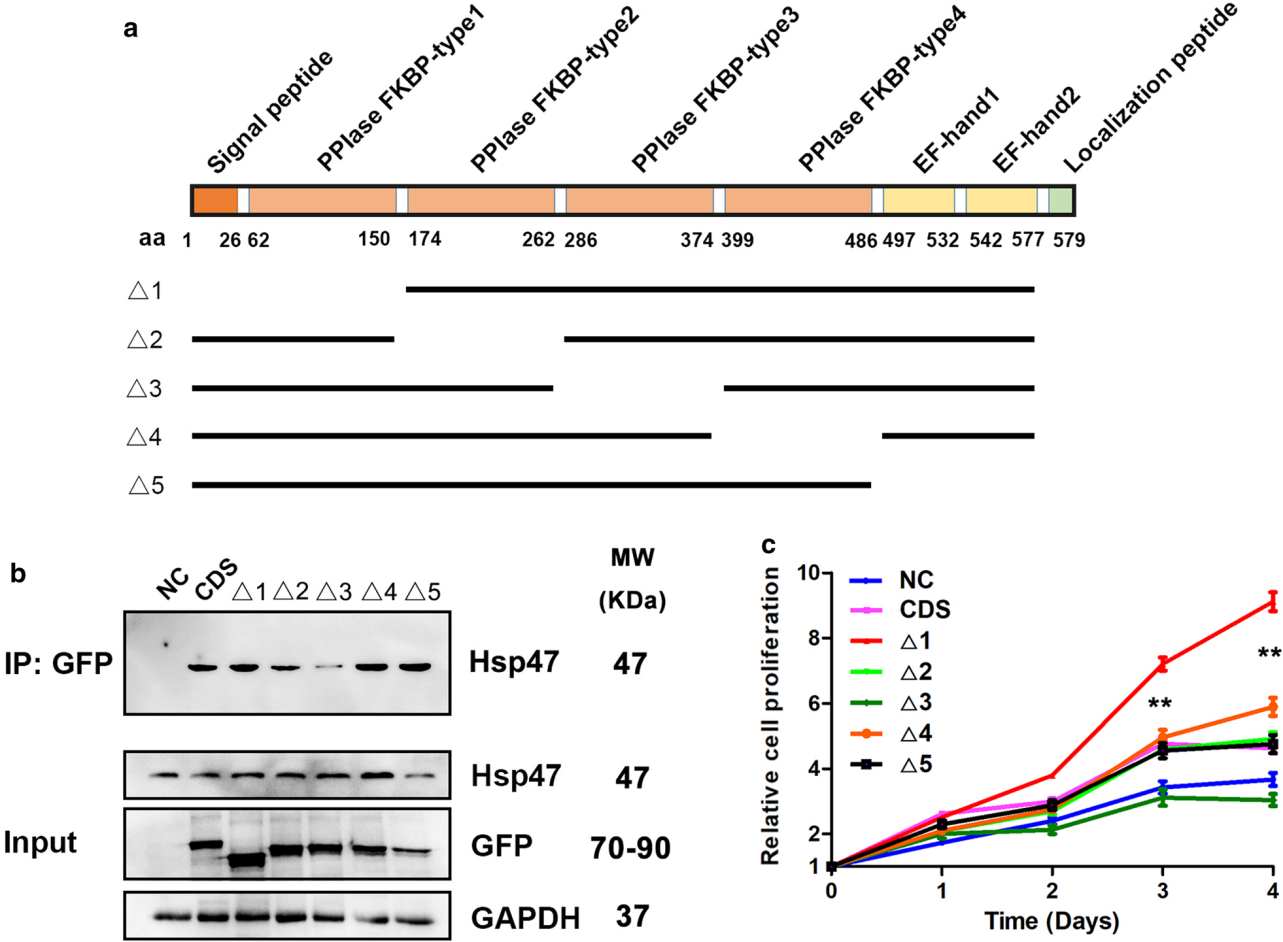
The FKBP-type3 PPIase domain is an active domain of FKBP10 interacting with Hsp47. **a** A diagram of five FKBP10 deletion mutants. **b** Co-IP and western blot analysis in shFKBP10 LN229 cells transfected with empty vector (NC), full length CDS and five mutants. **c** CCK-8 assay showed that the proliferation ability of seven types cells. The data are represented as means ± SD

FKBP10 contains several domains, including signal peptide, FKBP-type 1–4 PPIase, EF hand 1, EF hand 2 and localization peptide. But it remains unclear which domain interacts with Hsp47 and promotes the proliferation of glioma cells. Therefore, we constructed five FKBP10 domain-deletion mutants, FKBP10-Δ1 (deletion of signal peptide, FKBP-type 1 PPIase), FKBP10-Δ2 (deletion of FKBP-type 2 PPIase), FKBP10-Δ3 (deletion of FKBP-type 3 PPIase), FKBP10-Δ4 (deletion of FKBP-type 4 PPIase) and FKBP10-Δ5 (deletion of EF hand 1–2 and localization peptide) (Fig. 5a).

The result of co-immunoprecipitation and Western-blotting confirmed FKBP10-Δ3 mutant hard to interact with Hsp47 (Fig. 5b). Besides, CCK-8 assay showed that the decreased proliferation ability of shFKBP10 LN229 cells was not rescued when transfected with FKBP10-Δ3 mutant, whereas shFKBP10 LN229 cells transfected by other mutants or FKBP10-CDS with the elevated proliferation ability (Fig. 5c). These data revealed that the FKBP-type3 PPIase domain has an essential role in interacting with Hsp47 and boosting the proliferation of glioma cells.

### CREB regulates PCNA transcript process

**Fig. 6:**
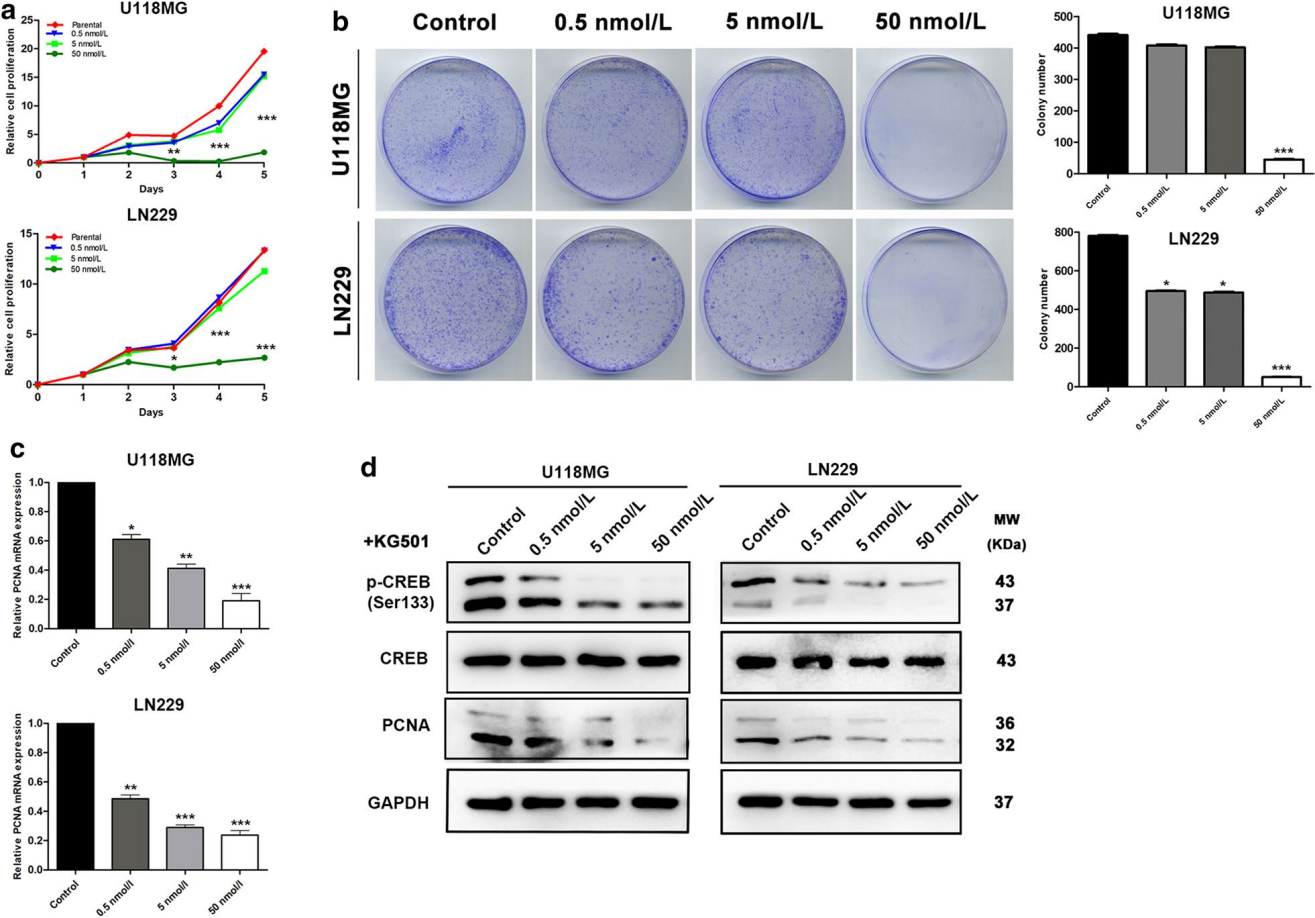
CREB regulates PCNA transcript process. **a**, **b** The CCK-8 cell proliferation tests and colony formation assays in U118MG and LN229 cells treated with 0.5, 5, 50 nmol/L of KG501. **c**, **d** The expression level of PCNA mRNA, PCNA protein in glioma cells treated with 0.5, 5, 50 nmol/L of KG501. The data are represented as means ± SD. *P < 0.05, **P < 0.01, ***P < 0.001

Previous studies showed that CREB is an essential transcription factor whose transcriptional effect is mdiated by its phosphorylation. The above results suggested that changes of CREB phosphorylation level was in accordance with that of PCNA mRNA after FKBP10 or Hsp47 knockdown. Thus, we further elucidated the regulating relationship between them. We found that inhibiting the activity of CREB with the inhibitor KG501 resulted in a significant decrease of cell proliferation rate, and colony number (Fig. 6a, b). And the decreased effect was in concentration-dependent manner. We also found that PCNA mRNA and protein expression level obviously downregulated following the decline of phosphorylated CREB (Fig. 6c, d). All these results indicated that phosphorylated CREB was involved in the transcript process of PCNA expression.

### Expression level of FKBP10 positively correlate with that of related molecules in subcutaneous xenografts and glioma tissues

**Fig. 7:**
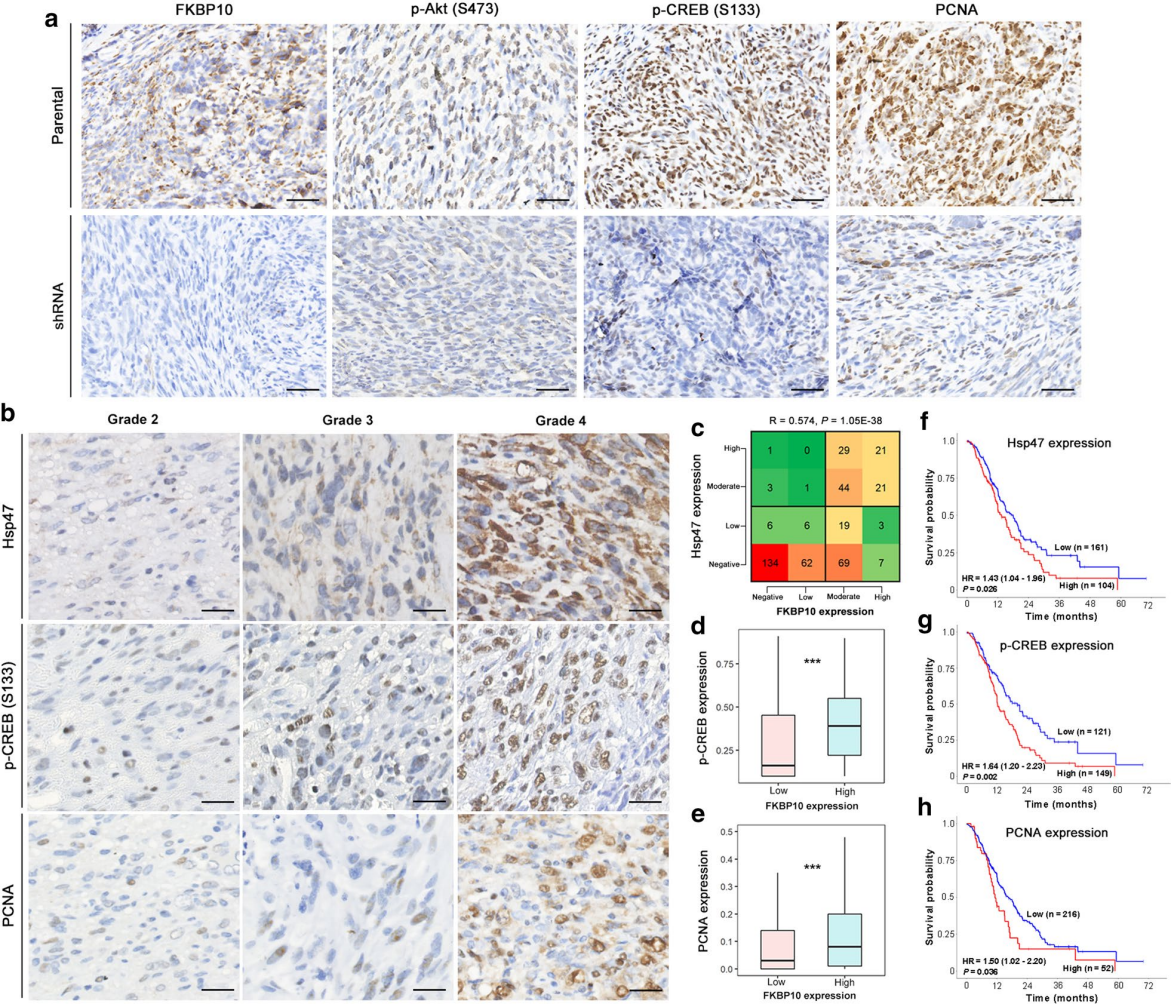
Expression level of FKBP10 related molecules in subcutaneous xenografts and glioma tissues. **a** Representative immunostaining images of FKBP10, p-AKT (Ser473), p-CREB (Ser133) and PCNA in FKBP10 parental and shRNA subcutaneous xenografts. Scale bar = 50 μm. **b** Representative immunostaining images of Hsp47, p-CREB (Ser133) and PCNA in grade 2, 3 and 4 gliomas. Scale bar = 25 μm. **c** Correlation analysis between FKBP10 expression and Hsp47 expression. **d**, **e** Expression of p-CREB (Ser133) and PCNA in high and low FKBP10 gliomas. **f**–**h** Survival analysis of Hsp47, p-CREB (Ser133) and PCNA overexpression in grade 4 glioma. ***P < 0.001

We detected FKBP10 and its downstream molecules by immunochemistry in subcutaneous xenografts. The immunostaining signal of FKBP10, p-AKT (Ser473), p-CREB (Ser133) and PCNA expression were significantly weakened in FKBP10 shRNA group compared to FKBP10 negative control group (Fig. 7a). The results of immunochemistry in glioma tissues displayed that positive immunostaining of Hsp47, CREB (Ser133) and PCNA were abserved in glioma tissues (Fig. 7b). Spearman correlation analysis between Hsp47 and FKBP10 revealed that these two molecules were strongly related (Fig. 7c, R = 0.574, *P* = 1.05E-38). Additionally, gliomas with high FKBP10 expression tend to own high expression level of p-CREB (Ser133) and PCNA (Fig. 7d, e, P = 1.57E-9 and 0.0009, respectively). Moreover, survival analysis showed that Hsp47, p-CREB (Ser133) and PCNA had significant inverse relationship with patients’ survival time (Fig. 7f–h, HR = 1.43, 95% CI 1.04–1.96, *P* = 0.026; HR = 1.64, 95% CI 1.20–2.23, *P* = 0.002; HR = 1.50, 95% CI 1.02–2.20, *P* = 0.036).

## Discussion

**Fig. 8:**
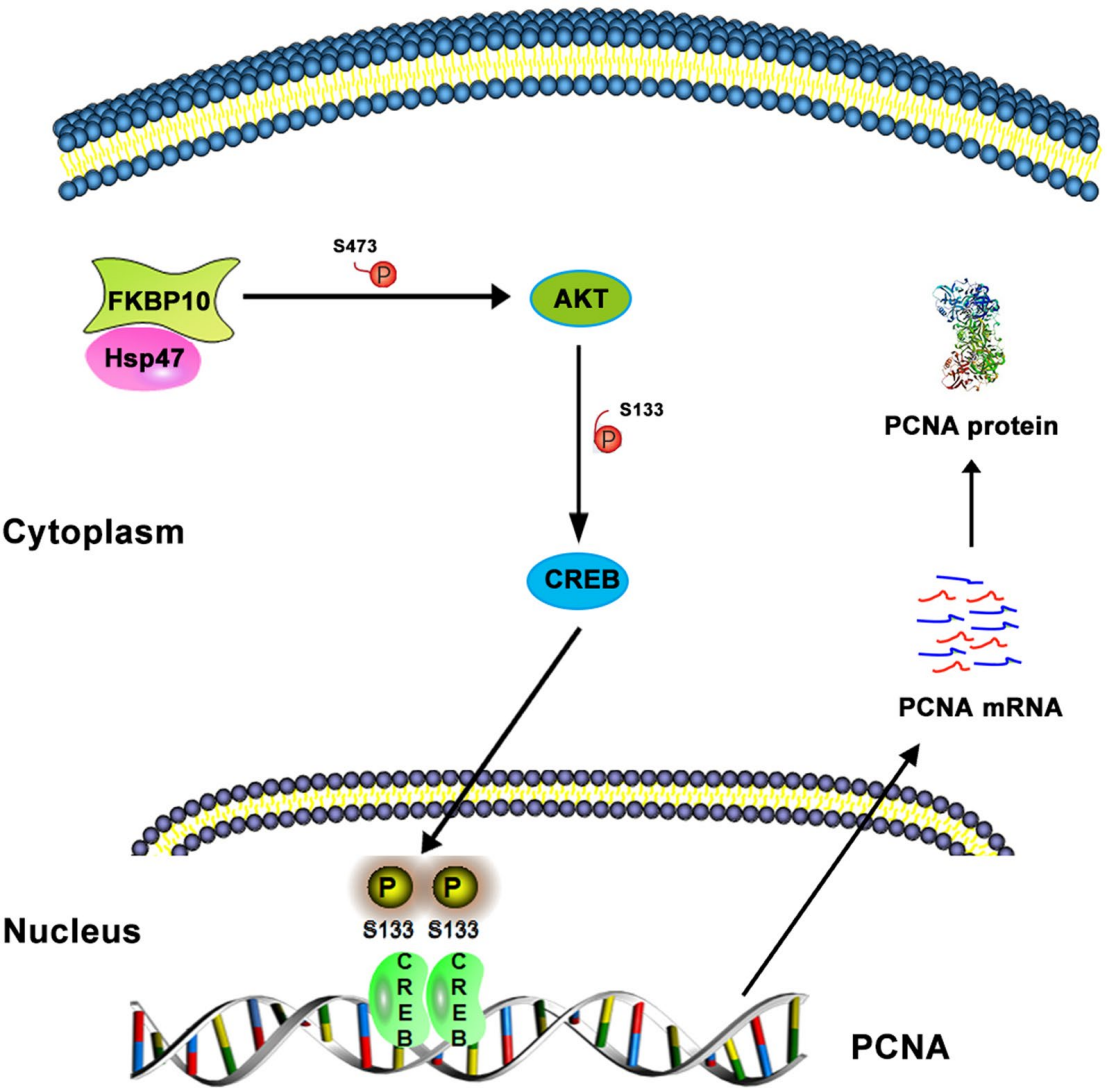
A schema diagrasm displaying the role of FKBP10 in regulating p-AKT, p-CREB and PCNA in glioma cells. Based on the findings of the present study, FKBP10 could promote PCNA expression and proliferation by interacting with Hsp47 and subsequently activating AKT-CREB pathway

An increasing number of molecular biomarkers, such as TERT promoter mutation, IDH1/2 mutation and 1p/19q co-deletion, have been used in clinic of glioma. However, the values of these biomarkers are mainly on the subtyping and prognostic prediction of the disease. Herein, we first demonstrate that FKBP10 is probably a potential target for the therapy of glioma. We found that FKBP10 was highly expressed in cancerous tissues, positively related to tumor grade and poor prognosis of patients. Especially, we observed that FKBP10, via interacting with Hsp47, increased the phosphorylation level of AKT at Ser473 and CREB at Ser133, which promoted the transcription of PCNA and the proliferation of glioma cells (Fig. 8). Our data suggested that targeting FKBP10-associated signaling might be a promising strategy in the treatment of glioma.

It has been shown that FKBP10 is abnormally expressed in several types of tumors. Olesen et al. found that FKBP10 was overexpressed not only in colorectal cancer (CRC) but also in precancerous lesions consisting of hyperplastic polyps, benign tubular adenomas, or tubulo-villous adenomas from the same patients, suggesting a possible involvement of FKBP10 in CRC genesis [21]. FKBP10 was also found to be upregulated in KRAS-mutant lung adenocarcinoma, renal cell carcinoma, and gastric cancer, and knockdown of FKBP10 is sufficient to hinder proliferation of tumor cells growth [18–20]. In coincidence with these observations, our study showed that FKBP10 highly expressed in glioma tissues, and knockdown of FKBP10 could inhibit the proliferation of glioma cells both in vitro and in vivo. It is worthwhile to mention that FKBP10 has been reported to be underexpressed in high-grade serous carcinomas (HGSC) and absence of FKBP10 expression was strongly related to prolonged survival time of patients, which suggest FKBP10 may play a role of tumor suppressor in HGSC [23]. Thus, FKBP10 may act as tumor promoter or suppressor in the process of tumor genesis and development depending on the cancer type.

Hsp47 protein encoded by *SERPINH1* gene localizes in endoplasmic reticulum (ER) and forms a chaperone complex together with FKBP10, which promotes the synthesis of type I procollagen in fibroblasts [11, 24]. Duran et al. found that Hsp47 could regulate FKBP10 protein level, while FKBP10 did not affect Hsp47 expression in fibroblast cells [25]. In the present study, we firstly detected FKBP10 interacting with Hsp47 in glioma cells and concomitant expression between FKBP10 and Hsp47 in glioma tissues. However, we did not find regulatory relationship between FKBP10 and Hsp47 expression, suggesting that the mechanisms underlying the roles of these two proteins are different in fibroblast and glioma cells. FKBP10 has eight domains including four FKBP domains, two EF-hands, one signal peptide and one ER locating domains. Several previous studies found that FKBP10 mutations disrupting the amino acid sequence of FKBP-type3 PPIase domain were a cause of recessive osteogenesis imperfecta and Bruck syndrome [26, 27], which suggested that FKBP-type3 PPIase domain may paly a key role in the biological function of FKBP10. In the present study, we identified for the first time that FKBP10 interacts Hsp47 by FKBP-type3 PPIase domain in glioma cells. It would be interesting to further investigate whether this interaction might be used as a target, or in combination with existing drug regimens, to improve the treatment of glioma. Temozolomide is the most commonly used chemotherapy drug for glioma. To date, there was no information concerning the relationship between the interaction of FKBP10/Hsp47 and chemotherapy. Future studies should explore whether abolishing the interaction of FKBP10/Hsp47 could enhance the sensitivity to TMZ in the therapy of glioma.

PCNA, by forming transcription initiation complex with other molecules such as DNA polymerase δ, promotes DNA synthesis in the proliferation process of eukaryotic cells [28, 29]. It has been reported that in glioma cells, CREB is constitutively activated, and PCNA is co-regulated by both CREB-dependent and -independent mechanisms [30, 31]. CREB is a key signaling molecule downstream of AKT and an essential transcription factor whose transcriptional effect is mediated by its phosphorylation [31, 32]. In the present study, we observed that PCNA was highly expressed and positively related to the level of p-CREB in glioma tissues. Also, we found a region in 200 bp upstream of the transcription initiation point of *PCNA* gene contains the binding site (CRE) of CREB, suggesting that p-CREB could upregulate PCNA expresson via promoting the transcript process. Accordingly, our results of in vivo experiments indicated that inhibition of p-CREB activity suppressed the proliferation of glioma cells.

Furthermore, in view of the overexpression and interaction of FKBP10 and Hsp47 as well as the effect of FKBP10 on the proliferation of glioma cells, the present study might provide potential targets for the therapy of the disease. Nintedanib was a multi-kinase inhibitor approved for idiopathic pulmonary fibrosis (IPF) therapy. It has been demonstrated that Nintedanib significantly down-regulated the protein level of FKBP10 in IPF fibroblasts [17]. On the other hand, polymers containing chemotherapy agents and Hsp47-binding peptide sequence have favorable inhibition effect on tumor for intracellular release of agents after specific binding with Hsp47 [33]. Thus, it will be important to explore the targeted therapy of glioma based on the alterations of FKBP10 and Hsp47.

## Conclusions

We showed for the first time that FKBP10 is overexpressed in glioma and involved in proliferation of glioma cells by interacting with Hsp47 and activating AKT-CREB-PCNA signaling pathways. Our findings suggest that inhibition of FKBP10 related signaling might offer a potential therapeutic option for glioma patients.

## Supplementary information

**Additional file 1: Table S1.** Baseline information of selected gliomas.

**Additional file 2: Figure S1.** FKBP10 and Hsp47 had no regulatory relationship with each other. **a** Western blot analysis assays of Hsp47 expression in 10 glioma cell lines. **b**, **c** Expression level of Hsp47 and FKBP10 in FKBP10 silencing and Hsp47 silencing glioma cells, respectively.

## Data Availability

The datasets used and analyzed during the current study are available from the corresponding author on reasonable request.

## Abbreviations

FKBP10: FK506 binding protein 10
Hsp47: Heat shock protein 47
CREB: CAMP-response element binding protein
PCNA: Proliferating cell nuclear antigen
IHC: Immunohistochemistry
TERT: Telomerase reverse transcriptase
IDH: Isocitrate dehydrogenase

## References

[1] Ostrom QT, Gittleman H, Xu J (2016). CBTRUS statistical report: primary brain and other central nervous system tumors diagnosed in the United States in 2009–2013. Neuro Oncol.

[2] Stupp R, Dietrich PY, Ostermann Kraljevic S (2002). Promising survival for patients with newly diagnosed glioblastoma multiforme treated with concomitant radiation plus temozolomide followed by adjuvant temozolomide. J Clin Oncol.

[3] Stupp R, Mason WP, van den Bent MJ (2005). Radiotherapy plus concomitant and adjuvant temozolomide for glioblastoma. N Engl J Med.

[4] Miller KD, Siegel RL, Lin CC (2016). Cancer treatment and survivorship statistics, 2016. CA Cancer J Clin.

[5] Bonner JM, Boulianne GL (2017). Diverse structures, functions and uses of FK506 binding proteins. Cell Signal.

[6] De Leon JT, Iwai A, Feau C (2011). Targeting the regulation of androgen receptor signaling by the heat shock protein 90 cochaperone FKBP52 in prostate cancer cells. Proc Natl Acad Sci U S A.

[7] Hsu FF, Chou YT, Chiang MT (2019). Signal peptide peptidase promotes tumor progression via facilitating FKBP8 degradation. Oncogene.

[8] Mange A, Coyaud E, Desmetz C (2019). FKBP4 connects mTORC2 and PI3K to activate the PDK1/Akt-dependent cell proliferation signaling in breast cancer. Theranostics.

[9] Zhu W, Li Z, Xiong L (2017). FKBP3 promotes proliferation of non-small cell lung cancer cells through regulating Sp1/HDAC2/p27. Theranostics.

[10] Luo K, Li Y, Yin Y (2017). USP49 negatively regulates tumorigenesis and chemoresistance through FKBP51-AKT signaling. EMBO J.

[11] Ishikawa Y, Holden P, Bachinger HP (2017). Heat shock protein 47 and 65-kDa FK506-binding protein weakly but synergistically interact during collagen folding in the endoplasmic reticulum. J Biol Chem.

[12] Gjaltema RA, van der Stoel MM, Boersema M (2016). Disentangling mechanisms involved in collagen pyridinoline cross-linking: The immunophilin FKBP65 is critical for dimerization of lysyl hydroxylase 2. Proc Natl Acad Sci U S A.

[13] Lietman CD, Lim J, Grafe I (2017). Fkbp10 deletion in osteoblasts leads to qualitative defects in bone. J Bone Miner Res.

[14] Xu XJ, Lv F, Liu Y (2017). Novel mutations in FKBP10 in Chinese patients with osteogenesis imperfecta and their treatment with zoledronic acid. J Hum Genet.

[15] Knuppel L, Heinzelmann K, Lindner M (2018). FK506-binding protein 10 (FKBP10) regulates lung fibroblast migration via collagen VI synthesis. Respir Res.

[16] Staab-Weijnitz CA, Fernandez IE, Knuppel L (2015). FK506-binding protein 10, a potential novel drug target for idiopathic pulmonary fibrosis. Am J Respir Crit Care Med.

[17] Knuppel L, Ishikawa Y, Aichler M (2017). A novel antifibrotic mechanism of nintedanib and pirfenidone. Inhibition of collagen fibril assembly. Am J Respir Cell Mol Biol..

[18] Liang L, Zhao K, Zhu JH (2019). Comprehensive evaluation of FKBP10 expression and its prognostic potential in gastric cancer. Oncol Rep.

[19] Ge Y, Xu A, Zhang M (2017). FK506 binding protein 10 is overexpressed and promotes renal cell carcinoma. Urol Int..

[20] Ramadori G, Konstantinidou G, Venkateswaran N (2015). Diet-induced unresolved ER stress hinders KRAS-driven lung tumorigenesis. Cell Metab.

[21] Olesen SH, Christensen LL, Sorensen FB (2005). Human FK506 binding protein 65 is associated with colorectal cancer. Mol Cell Proteomics.

[22] Cai HQ, Wang PF, Zhang HP (2018). Phosphorylated Hsp27 is mutually exclusive with ATRX loss and the IDH1(R132H) mutation and may predict better prognosis among glioblastomas without the IDH1 mutation and ATRX loss. J Clin Pathol.

[23] Quinn MC, Wojnarowicz PM, Pickett A (2013). FKBP10/FKBP65 expression in high-grade ovarian serous carcinoma and its association with patient outcome. Int J Oncol.

[24] Duran I, Martin JH, Weis MA (2017). A chaperone complex formed by HSP47, FKBP65, and BiP modulates telopeptide lysyl hydroxylation of type i procollagen. J Bone Miner Res.

[25] Duran I, Nevarez L, Sarukhanov A (2015). HSP47 and FKBP65 cooperate in the synthesis of type I procollagen. Hum Mol Genet.

[26] Trancozo M, Moraes MVD, Silva DA (2019). Osteogenesis imperfecta in Brazilian patients. Genet Mol Biol.

[27] Kelley BP, Malfait F, Bonafe L (2011). Mutations in FKBP10 cause recessive osteogenesis imperfecta and Bruck syndrome. J Bone Miner Res..

[28] Kowalska E, Bartnicki F, Fujisawa R (2018). Inhibition of DNA replication by an anti-PCNA aptamer/PCNA complex. Nucleic Acids Res..

[29] Boehm EM, Gildenberg MS, Washington MT (2016). The many roles of PCNA in eukaryotic DNA replication. Enzymes.

[30] Barresi V, Mondello S, Branca G (2015). p-CREB expression in human gliomas: potential use in the differential diagnosis between astrocytoma and oligodendroglioma. Hum Pathol.

[31] Daniel P, Filiz G, Brown DV (2014). Selective CREB-dependent cyclin expression mediated by the PI3K and MAPK pathways supports glioma cell proliferation. Oncogenesis.

[32] Xiao X, Li BX, Mitton B (2010). Targeting CREB for cancer therapy: friend or foe. Curr Cancer Drug Targets.

[33] Duarte BDP, Bonatto D (2018). The heat shock protein 47 as a potential biomarker and a therapeutic agent in cancer research. J Cancer Res Clin Oncol.

