# Supplementary material for: FKBP10 promotes proliferation of glioma cells via activating AKT-CREB-PCNA axis
Source: J Biomed Sci. 2021 Feb 9;28:13. doi: 10.1186/s12929-020-00705-3 (PMC7871608; doi:10.1186/s12929-020-00705-3)
Supplement: Supplementary file 1 — Additional file 1: Table S1. Baseline information of selected gliomas. [file 12929_2020_705_MOESM1_ESM.docx]

**Supplementary Table S1. Baseline information of selected gliomas.**

| Variable | No.(%) |
| --- | --- |
| Gender |  |
| Male | 254 (59.1%) |
| Female | 176 (40.9%) |
| Age (years) |  |
| ≤60 | 319 (74.2%) |
| >60 | 111 (25.8%) |
| KPS |  |
| <70 | 87 (20.2%) |
| >=70 | 220 (51.2%) |
| NA | 123 (28.6%) |
| Grade |  |
| 2 | 74 (17.2%) |
| 3 | 70 (16.3%) |
| 4 | 286 (66.5%) |
| Radiaton/chemotherapy |  |
| Yes | 291 (67.7%) |
| No | 139 (32.3%) |
