# Supplementary figures and images for: FKBP10 promotes proliferation of glioma cells via activating AKT-CREB-PCNA axis
Source: J Biomed Sci. 2021 Feb 9;28:13. doi: 10.1186/s12929-020-00705-3 (PMC7871608; doi:10.1186/s12929-020-00705-3)

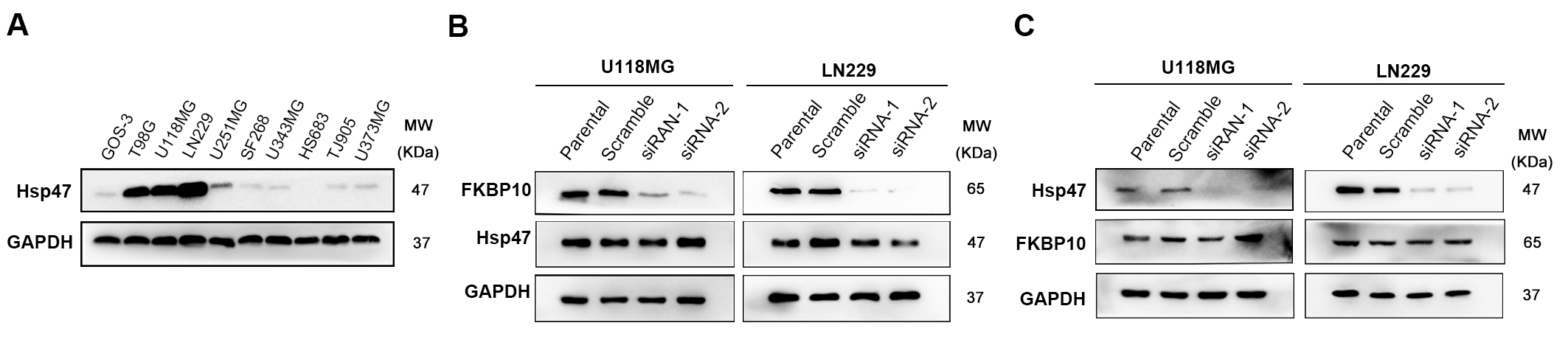

Supplement: Supplementary file 2 — Additional file 2: Figure S1. FKBP10 and Hsp47 had no regulatory relationship with each other. a Western blot analysis assays of Hsp47 expression in 10 glioma cell lines. b, c Expression level of Hsp47 and FKBP10 in FKBP10 silencing and Hsp47 silencing glioma cells, respectively. [file 12929_2020_705_MOESM2_ESM.jpg]
